# Supplementary material for: EpoR Activation Stimulates Erythroid Precursor Proliferation by Inducing Phosphorylation of Tyrosine-88 of the CDK-Inhibitor p27Kip1
Source: Cells. 2023 Jun 23;12(13):1704. doi: 10.3390/cells12131704 (PMC10340229; doi:10.3390/cells12131704)
Supplement: Supplementary file 1 [file cells-12-01704-s001.zip › cells-2356854-supplementary.pdf]

Supplementary Data

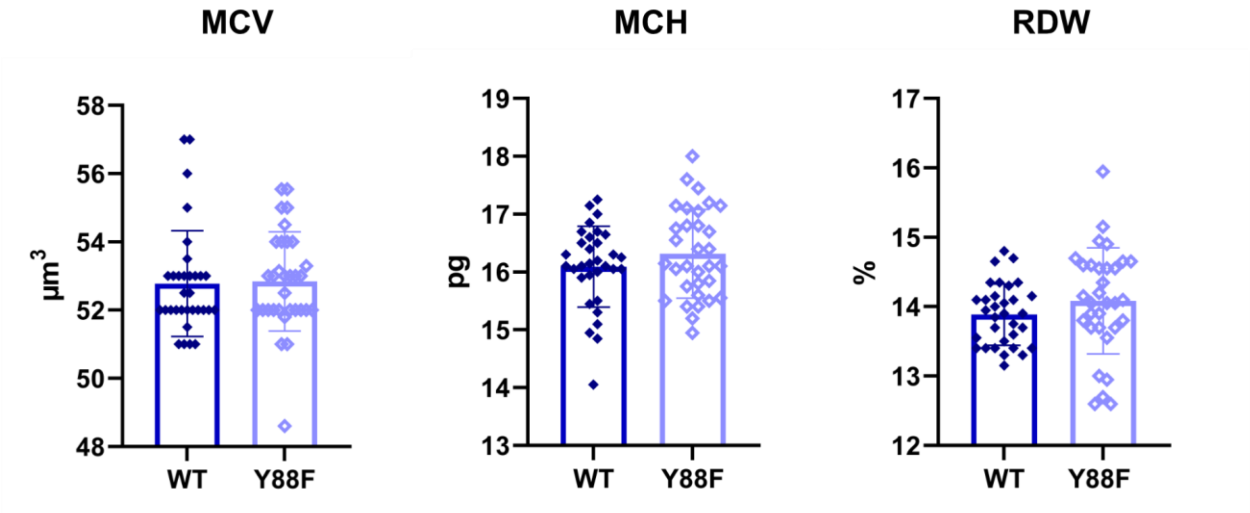

Figure S1. Peripheral blood analysis of 7-8 weeks old male wild type and knock-in mice with the use of the ABC blood counter. No significant differences were observed in the values of MCV (Mean Corpuscular Volume), MCH (Mean Corpuscular Hemoglobin) and RDW (Red Cell Distribution Width) between WT and p27Y88F knock-in mice. $p > 0.05$, unpaired t-test. (WT n=32, KI n=32)

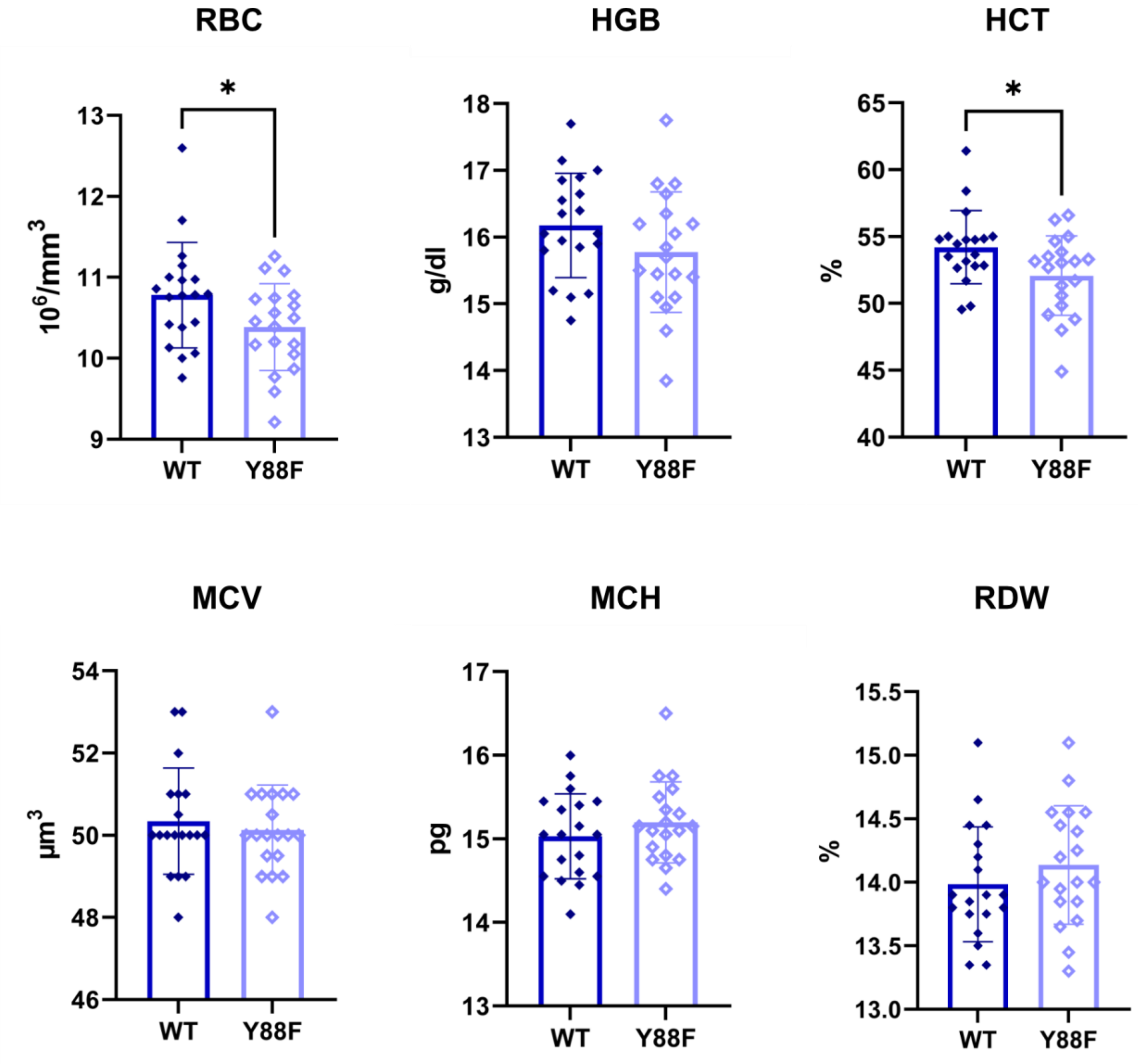

Figure S2: Analysis of red blood cell parameters uncovers reduced red blood cell count and hematocrit levels in 22-24 weeks old p27-Y88F knock-in mice. Red blood cell (RBC) count ($p<0.05$, unpaired t test), hemoglobin (HGB), hematocrit levels (HCT) ($p<0.05$,unpaired t test), mean corpuscular volume (MCV), mean corpuscular hemoglobin (MCH) and red cell distribution width (RDW) of 22-24 weeks old male WT and $p27^{Y88F/Y88F}$ mice. Blood values were measured with the scil Vet ABC Analyzer. WT n=19, KI n=19.

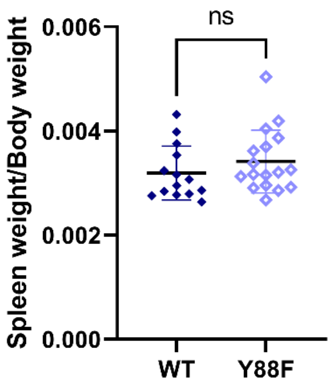

Figure S3. Spleen to body weight ratio of 7 weeks old WT and KI mice. $p > 0.05$, unpaired t-test. (WT n=14, KI n=17)
